# ﻿Morphology, phylogeny, mitogenomics and metagenomics reveal a new entomopathogenic fungus *Ophiocordycepsnujiangensis* (Hypocreales, Ophiocordycipitaceae) from Southwestern China

**DOI:** 10.3897/mycokeys.94.89425

**Published:** 2022-12-21

**Authors:** Tao Sun, Weiqiu Zou, Quanying Dong, Ou Huang, Dexiang Tang, Hong Yu

**Affiliations:** 1 Yunnan Herbal Laboratory, College of Ecology and Environmental Sciences, Yunnan University, Kunming 650504, Yunnan, China Yunnan University Kunming China; 2 School of Life Sciences, Yunnan University, Kunming 650504, Yunan, China School of Ecology and Environmental Science Kunming China

**Keywords:** microbial community, mitochondrial genome, new species, *Ophiocordycepsnujiangensis*, phylogenetic analyses

## Abstract

*Ophiocordyceps* contains the largest number of *Cordycepssensu lato*, various species of which are of great medicinal value. In this study, a new entomopathogenic fungus, *Ophiocordycepsnujiangensis*, from Yunnan in southwestern China, was described using morphological, phylogenetic, and mitogenomic evidence, and its fungal community composition was identified. It was morphologically characterized by a solitary, woody, and dark brown stromata, smooth-walled and septate hyphae, solitary and gradually tapering conidiogenous cells with plenty of warty protrusions, and oval or fusiform conidia (6.4–11.2 × 3.7–6.4 µm) with mucinous sheath. The phylogenetic location of *O.nujiangensis* was determined based on the Bayesian inference (BI) and the maximum likelihood (ML) analyses by concatenating nrSSU, nrLSU, *tef-1a*, *rpb1*, and *rpb2* datasets, and ten mitochondrial protein-coding genes (PCGs) datasets (*atp6*, *atp9*, *cob*, *cox2*, *nad1*, *nad2*, *nad3*, *nad4*, *nad4L*, and *nad5*). Phylogenetic analyses revealed that *O.nujiangensis* belonged to the *Hirsutellasinensis* subclade within the *Hirsutella* clade of *Ophiocordyceps*. And *O.nujiangensis* was phylogenetically clustered with *O.karstii*, *O.liangshanensis*, and *O.sinensis*. Simultaneously, five fungal phyla and 151 fungal genera were recognized in the analysis of the fungal community of *O.nujiangensis*. The fungal community composition differed from that of *O.sinensis*, and differences in the microbial community composition of closely related species might be appropriate as further evidence for taxonomy.

## ﻿Introduction

The genus *Ophiocordyceps* was introduced by Petch (1931), with *O.blattae* Petch as the type. This genus accommodated species with features of head-cover asci, septate and non-disarticulating ascospores (Petch 1931). Then, the genus was regarded as a subgenus of *Cordyceps* (Kobayasi 1941, 1982; Mains 1958). Until 2007, Sung et al. (2007) erected a new family Ophiocordycipitaceae based on phylogenetic analysis and the characteristics of darkly pigmented stromata, which were pliant to wiry or fibrous to tough in texture. And they revised the classification of *Ophiocordyceps*, treating it as the type genus of Ophiocordycipitaceae. *Ophiocordyceps* has the largest number of species in Ophiocordycipitaceae, with 307 species named in Ophiocordyceps to date. (http://www.indexfungorum.org/, retrieval on November 3, 2022).

The methods of morphology and phylogeny were utilized for species identification, and the phylogenetic analyses based on concatenating nrSSU, nrLSU, *tef-1*α, *rpb1*, and *rpb2* datasets became the popular means (Sung et al. 2007; Quandt et al. 2014; Sanjuan et al. 2015; Wang et al. 2020a). Moreover, the mitochondrial genome had been an effective instrument for studying species’ origin, classification, and evolution due to its advantages of high copy number, low mutation rate, and fast evolution rate (Alexeyev et al. 2013; Aguileta et al. 2014; Williams et al. 2014). The significant difference in the mitochondrial genome of fungi could be distinguished (Nie et al. 2019). The biogenetic analyses of the fungal mitochondrial genome could verify the genetically related species. NCBI has published the mitochondrial genomes of more than 680 fungi, including approximately 60 species of Hypocreales (Chen et al. 2021; Zhao et al. 2021).

Some species in *Ophiocordyceps* have enormous medicinal and commercial value, such as *O.sinensis*, traditional in Chinese medicine. Owing to their extraordinary efficacy, wild sources were widely sold as commodities and gradually became scarce. (Han et al. 2019; Dai et al. 2020). Therefore, seeking additional new resources would defuse the tense situation. For example, *O.lanpingensis* and *O.xuefengensis* had been authenticated as possessing ingredients that were beneficial for health and considered to be desirable alternatives for *O.sinensis* (Zou et al. 2017; Zhang et al. 2017). *Ophiocordyceps* is widely distributed in China, and of particular note are some recent reports of new species from southwestern China (Wang et al. 2018; Wang et al. 2020b; Chen et al. 2021).

The companion fungi were essential for the growth and development of the host. For example, *Tuber*-associated microbial communities played a potentially important role in mycelial growth, ascocarp development, and mycorrhizal synthesis of *Tuber* (Li et al. 2018). And adding *Grifola* sp. in the cultivation process of *G.umbellate* could promote sclerotia formation (Guo et al. 2002). Thus, the composition and diversity of companion fungi should be analyzed to gain insight into new species and their microbial resources.

In this study, a new species of *Ophiocordyceps*, which parasitized on the larvae of Hepialidae, was collected from Yunnan in southwestern China. The phylogenetic location was elucidated based on the Bayesian inference (BI) and the maximum likelihood (ML) analyses by concatenating nrSSU, nrLSU, *tef-1a*, *rpb1*, and *rpb2* datasets, and mitochondrial protein-coding genes (PCGs) datasets. Morphological characteristics were observed and recorded. The composition and diversity of the fungal communities hosting the new species were identified.

## ﻿Methods

### ﻿Sample collection and isolation

Samples were collected on Hepialidae larvae in the soil in Yajiaoluo (27°07'48"N, 98°52'12"E), Fugong County, Nujiang Prefecture, Yunnan Province, China. Specimens were photographed in the fields with a Canon 750D digital camera. The fresh specimens were placed into the sterile culture dish, then transferred to the laboratory and deposited in the Yunnan Herbal Herbarium (**YHH**), Yunnan University.

Specimens were isolated and cultured using the tissue isolating method (Yin and Zhang 2015; Wang et al. 2020b) as follows. Specimens were dipped into 75% alcohol for 2 min to sterilize the surface and then washed with sterile water. The 2–3 mm sclerotium was ripped by tweezers and put on the culture medium (200 g potato, 20 g dextrose 20, 15–20 g agar, 10 g yeast extract, 5 g peptone in 1 L sterile water) (Xu et al. 2019), with three replications. Then they were transferred to the room at 25 °C for culturing. The cultures were deposited in the Yunnan Fungal Culture Collection (**YFCC**), at Yunnan University.

### ﻿Morphological observations

A moderate quantity of pure cultures was picked by an inoculating needle onto the center of the culture medium and maintained at 25 °C. After 6–10 weeks, shape, size, texture, and color were photographed with a Canon 750D camera. The superficial pure cultures were lightly stuck on transparent adhesive tapes, then the tapes were patched on slides, and the slides were placed on the Olympus BX53 microscope for micro-morphological observations and measurements (Wang et al. 2020a; Wang et al. 2020b).

### ﻿DNA extraction, PCR amplification, and sequencing of nuclear genes

The genomic DNA of the samples (containing specimens and pure cultures) was isolated using the ZR Fungal DNA kit (Zymo, California, USA), then the DNA extract was checked on 1% agarose gel, and DNA concentration and purity were determined with NanoDrop ND-2000 spectrophotometer (Thermo Scientific, Wilmington, USA). The nrSSU and nrLSU (nuclear ribosomal small and large subunits), *rpb1* and *rpb2* (the largest and second-largest subunit sequences of RNA polymerase II), and *tef-1*α (the translation elongation factor 1α) regions were amplified with the primer pairs used by Wang et al. (2020b). The PCR mixtures contained 2 × Taq PCR Master Mix (Tiangen, Beijing, China) 25 μL, forward primer (10 μM) 0.5 μL, reverse primer (10 μM) 0.5 μL, template DNA (1 ng/μL) 1 μL, and finally added sterile ddH_2_O up to 50 μL. Finally, the PCR amplification and sequencing were performed as described by Wang et al. (2015).

### ﻿Sequencing, assembly, and annotation of mitogenome

The genomic DNA of the pure cultures was isolated through the above-mentioned method, the extracted DNA was transported to BGI genomics Co., Ltd (Wuhan, China) for sequencing. The sequencing library was built by the IlluminaTruseq DNA Sample Preparation Kit (BGI, Shenzhen, China), and the Illumina HiSeq 4000 Platform was applied to the PE2 × 150 bp sequencing. After data quality control, the unpaired, short, and low-quality reads were removed, and the clean reads were obtained (Zhao et al. 2021). Next, the reads of the mitogenome were collected from the clean data employing GetOrganelle v.1.6.2e, and the mitogenome was assembled using BLAST 2.2.30 and SPAdes. V.3.13.0. The mitogenome was initially annotated by MFannot (https://megasun.bch.umontreal.ca/RNAweasel/, accessed on 10 December 2020) and MITOS (http://mitos2.bioinf.uni-leipzig.de/index.py, accessed on 10 December 2020) (Valach et al. 2014; Jin et al. 2020; Chen et al. 2021).

### ﻿Phylogenetic analyses

For determining the phylogenetic location of the species, phylogenetic analyses were conducted with the combined sequence data of nrSSU, nrLSU, *rpb1*, *rpb2*, and *tef-1*α (Wang et al. 2015; Wang et al. 2020a; Wang et al. 2020b), and ten protein-coding genes (PCGs, *atp6*, *atp9*, *cob*, *cox2*, *nad1*, *nad2*, *nad3*, *nad4*, *nad4L*, *nad5*) of mitogenomes, respectively (Chen et al. 2021; Zhao et al. 2021). The Bayesian inference (BI) and the maximum likelihood (ML) methods were performed for the phylogenetic analyses by MrBayes v3.1.2 (Ronquist and Huelsenbeck 2003) and RaxML 7.0.3 (Stamatakis et al. 2008). The GTR + G + I model was determined by jModelTest version 2.1.4 (Darriba et al. 2012) with 10 million generations for the BI analysis. And the ML analysis was run with the GTR + I model on 10,000 rapid bootstrap replicates. *Tolypocladiuminflatum* W. Gams and *T.ophioglossoides* (J.F. Gmel.) C.A. Quandt, Kepler & Spatafora were designated as the outgroup taxa for the analysis of nrSSU, nrLSU, *rpb1*, *rpb2*, and *tef-1*α datasets. And *Penicilliumcitrinum* Thom and *Neurosporacrassa* Shear & B.O. Dodge were designated as the outgroup taxa for the analysis of 10 PCGs. The GenBank accession numbers of the 10 PCGs (*atp6*, *atp9*, *cob*, *cox2*, *nad1*, *nad2*, *nad3*, *nad4*, *nad4L*, and *nad5*) annotated from the specimen YFCC8894 were ON868828–ON868837.

### ﻿Isolation of total DNA, PCR amplification, and high-throughput sequencing

The microbial genomic DNA of the fruiting body from four different specimens (S1–S4) was isolated through the method mentioned above. The ITS (internal transcribed spacer) regions were amplified with primer pairs ITS5 (5’-GGAAGTAAAAGTCGTAACAAGG-3’) and ITS4 (5’-TCCTCCGCTTATTGATATGC-3’) (White et al. 1990), by an ABI GeneAmp 9700 PCR thermocycler (ABI, CA, USA). The PCR amplifications were performed as follows: initial denaturation at 95 °C for 3 min, followed by 27 cycles of denaturing at 95 °C for 30 s, annealing at 55 °C for 30 s and extension at 72 °C for 45 s, and single extension at 72 °C for 10 min. The PCR mixtures contained 5 × Fast Pfu buffer 4 μL, 0.4 μL Fast Pfu polymerase, forward primer (5 μM) 0.8 μL, reverse primer (5 μM) 0.8 μL, template DNA (1ng/μL) 10 μL, and finally added sterile ddH_2_O up to 20 μL. The PCR products were extracted from 2% agarose gel and purified by the AxyPrep DNA Gel Extraction Kit (Axygen Biosciences, Union City, USA) and quantified by Quantus Fluorometer (Promega, Madison, USA).

Purified amplicons were pooled in equimolar amounts and paired-end sequenced on an Illumina MiSeq PE300 platform (Illumina, San Diego, USA), following the standard protocols by Majorbio Bio-Pharm Technology Co. Ltd. (Shanghai, China). The raw reads were deposited into the NCBI Sequence Read Archive (SRA) database (Sequence Read Archive (SRA) Accession Number: SAMN28950406–SAMN28950409).

Raw FASTQ files were de-multiplexed using an in-house Perl script, and then quality-filtered by fastp version 0.19.6 (Chen et al. 2018) and merged by FLASH version 1.2.7 (Magoč and Salzberg 2011). Then the optimized sequences were clustered into operational taxonomic units (OTUs) employing UPARSE 7.1 (Edgar 2013) with the 97% sequence similarity level. Chimeric sequences, chloroplast sequences, mitochondrial sequences, and the OTUs identified as Plantae, Rhizaria, Chromista, and those with no rank and unclassified kingdom were removed from samples.

### ﻿Composition and phylogenetic analysis of microbial communities

Bioinformatic analysis was carried out by the Majorbio Cloud platform (https://cloud.majorbio.com). The taxonomy of each OTU representative sequence was analyzed by RDP Classifier version 2.2 (Wang et al. 2007) against the ITS gene database (Unite V7.2) through a confidence threshold of 0.7. A phylogenetic tree was constructed to illustrate the relationships between the fungi at the family level, employing FastTree version 2.1.3 (http://www.microbesonline.org/fasttree/) and the ML algorithm (Zhang et al. 2015).

## ﻿Results

### ﻿Phylogenetic analyses of nuclear genes

**Figure 1.**
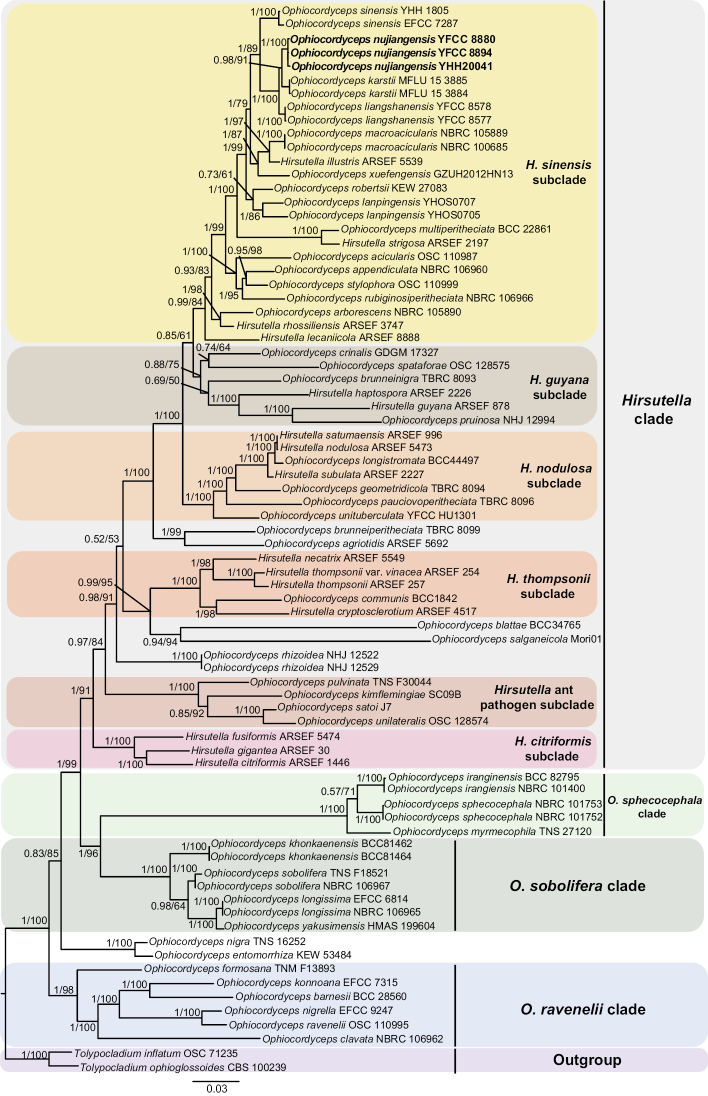
Phylogenetic placement of *Ophiocordycepsnujiangensis* inferred from the Bayesian inference (BI) and the maximum likelihood (ML) analyses by concatenating nrSSU, nrLSU, *tef-1a*, *rpb1*, and *rpb2* datasets. The BI posterior probabilities (≥ 0.5) and the ML bootstrap values (≥ 50%) were indicated at the nodes. The specimens analyzed in this study were shown in bold type.

The phylogenetic tree was built with the 72 taxa by the Bayesian inference (BI) and the maximum likelihood (ML) methods. *Tolypocladiuminflatum* OSC 71235 and *Tolypocladiumophioglossoides* CBS 100239 were designated as the outgroup taxa (Fig. 1; Suppl. material 1). The five-gene phylogenetic trees based on the BI and the ML analyses had similar topologies. The reconstructed phylogenetic tree of *Ophiocordyceps* contained four statistically well-supported clades. And the *Hirsutella* clade had six statistically well-supported subclades. It was similar to the analyses by Sanjuan et al. (2015), Simmons et al. (2015), and Wang et al. (2018). The three specimens of *Ophiocordycepsnujiangensis* (Wild sample YHH20041, pure cultures YFCC 8880, and YFCC 8894) were clustered together and formed a separate clade (the BI posterior probabilities = 1, the ML bootstrap = 98%). *O.nujiangensis* was closely related to *O.karstii*, *O.liangshanensis* and *O.sinensis* with strong support (Fig. 1). The similarities between the YFCC 8880 strain of *O.nujiangensis* and the most relevant were 99.66% (nrSSU), 99.87% (nrLSU), 98.53% (*tef-1*α), 98.53% (*rpb1*) and 98.80% (*rpb2*) in the BALST (The basic local alignment search tool) results of NCBI database. The BALST results of the YFCC 8894 strain were 99.87% (nrLSU), 98.52% (*tef-1*α), and 98.69% (*rpb1*). And the BALST results of sample YHH20041 were 100% (nrSSU), 99.87% (nrLSU), 98.64% (*tef-1*α), 98.36% (*rpb1*) and 98.66% (*rpb2*).

### ﻿Phylogenetic analyses of mitochondrial genes

**Figure 2.**
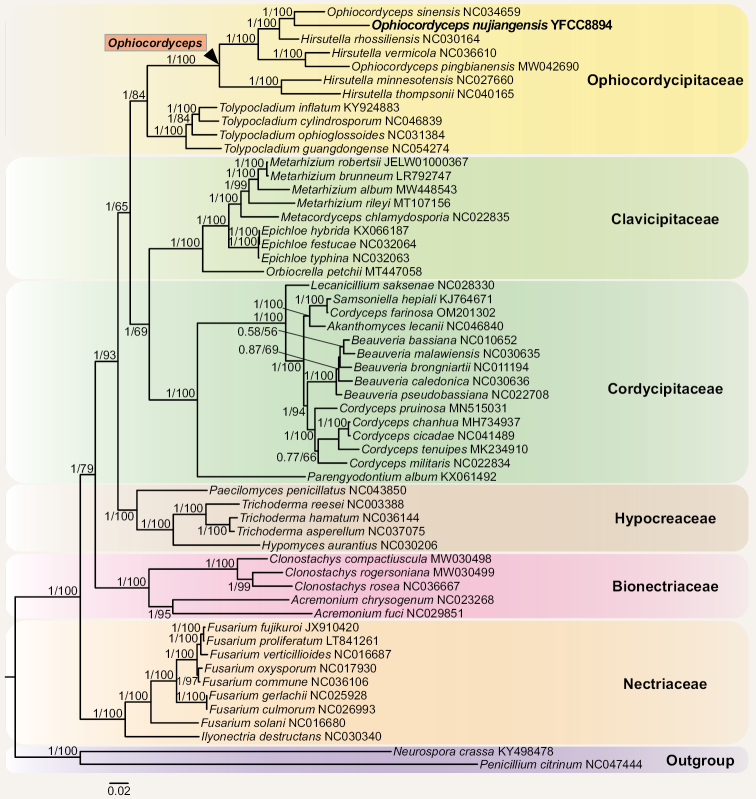
Phylogenetic tree of Hypocreales based on the Bayesian inference (BI) and the maximum likelihood (ML) analyses of 10 PCGs. The 10 PCG genes included *atp6*, *atp9*, *cob*, *cox2*, *nad1*, *nad2*, *nad3*, *nad4*, *nad4L* and *nad5*. The values at the nodes were the BI posterior probabilities and the ML bootstrap proportions, respectively. The specimen analyzed in this study was given in bold type.

The mitogenome of *O.nujiangensis* was assembled and annotated. And 10 PCGs (protein-coding genes) were chosen for the phylogenetic analyses, including 2 subunits of ATP synthase (*atp6* and *atp9*), 1 cytochrome b gene (*cob*), 1 subunit of cytochrome c oxidase (*cox2*), and 6 subunits of NADH dehydrogenase complex (*nad1*, *nad2*, *nad3*, *nad4*, *nad4L*, and *nad5*). The BI and the ML trees were estimated for phylogenetic analyses of Hypocreales based on the mitochondrial PCG dataset of 55 species from GenBank. *Penicilliumcitrinum* and *Neurosporacrassa* were designated as the outgroup taxa (Suppl. material 2). As shown in Figure 2, six well-supported clades were recognized in Hypocreales, namely Bionectriaceae, Clavicipitaceae, Cordycipitaceae, Hypocreaceae, Nectriaceae, and Ophiocordycipitaceae. And *Ophiocordycepsnujiangensis* was clustered collectively with *O.sinensis*, *H.rhossiliensis*, *H.vermicola*, *O.pingbianensis*, *H.minnesotensis*, and *H.thompsonii* in *Ophiocordyceps*. *O.nujiangensis* formed a separate clade (the BI posterior probabilities = 1, the ML bootstrap = 100%), and was also closely grouped with *O.sinensis* (Fig. 2).

### ﻿Taxonomy

#### ﻿ Ophiocordyceps nujiangensis

Taxon classificationFungiHypocrealesOphiocordycipitaceae

H. Yu, T. Sun & W.Q. Zou sp. nov.

EE5ECB8F-B9E9-51E5-AD79-4623AA096EC4

MB 844428

Fig. 3

##### Etymology.

Nujiangensis, referring to the collection site of this species, Nujiang.

##### Holotype.

Yajiaoluo, Fugong County, Nujiang Prefecture, Yunnan Province, China. 98°52.20'N, 27°07.80'E, alt 1980 m, on the larvae of Hepialidae in soil, 6 June 2021, Hong Yu (YHH 20039, holotype; YFCC 8880, ex-holotype culture).

**Figure 3.**
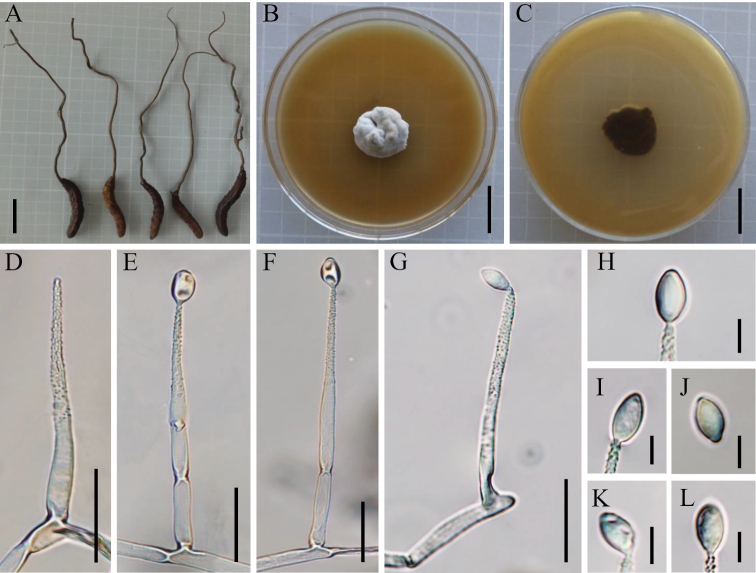
*Ophiocordycepsnujiangensis***A** intact wild material **B** colony obverse on PDA with peptone and yeast extract powder **C** colony reverse on PDA with peptone and yeast extract powder **D** conidiogenous cells **E–G** conidiogenous cells and conidia **H–L** conidia. Scale bars: 3 cm (**A**); 2 cm (**B, C**); 20 µm (**D–G**); 5 µm (**H–L**).

##### Sexual stage.

Stromata grew from the head of Hepialidae larva, solitary, certain branches at middle, gradually tapering from base to tip, woody, hard, dark brown (1545C, the number of PANTONE color, https://www.pantone.com), 14.8–18.2 cm long. Microscopic morphology to be determined.

##### Asexual stage.

*Hirsutella*. The colonies grew slowly on PDA, adding peptone (5 g/L) and yeast extract powder (10g/L) to PDA could accelerate the growth. Culturing at room temperature (16–20 °C) after 14 weeks, the colonies increased to 20–21 mm, hard, slight protuberance in the middle, pale gray (Cool gray 1 C), reverse black brown (Black 4 XGC). Hyphae hyaline, septate, smooth-walled. Conidiogenous cells hyaline, solitary, 54.9–76.5 (AVE = 50.50 ± 0.24) µm long, gradually tapering, base width 3.6–4.9 (AVE = 4.32 ± 0.11) µm, tip width 1.0–1.5 (AVE = 1.30 ± 0.11) µm, with warty protrusions from the middle to the top and more on the top, smooth-walled. Conidia hyaline, oval or fusiform, with smooth walls and mucinous sheath, 6.4–11.2 (AVE = 7.95 ± 0.15) × 3.7–6.4 (AVE = 4.73 ± 0.16) µm.

##### Host.

Larvae of Hepialidae.

##### Habitat.

Parasitized on Hepialidae larvae in the soil.

##### Distribution.

Yajiaoluo, Fugong County, Nujiang Prefecture, Yunnan Province, China.

##### Other material examined.

Yajiaoluo, Fugong County, Nujiang Prefecture, Yunnan Province, China. 98°52.20'N, 27°07.80'E, alt 1980 m, on the larvae of Hepialidae in soil, 6 June 2021, Hong Yu (YHH20040, YFCC 8894; YHH 20041).

##### Notes.

*Ophiocordycepsnujiangensis* was closely phylogenetically related to *O.karstii* and *O.liangshanensis*. The formation of stromata on the head of the host was a feature common to all three species. However, the length of the stromata varies between the three species. *O.nujiangensis* had a stromata length longer than *O.karstii*, but shorter than *O.liangshanensis* (Table 1). *O.nujiangensis*, on the other hand, had slightly longer conidiophores and slightly smaller conidia than *O.liangshanensis* (Table 1).

**Table 1.** A morphological comparison of *Ophiocordycepsnujiangensis* and its allies.

Species	Host	Stromata	Ascomata	Asci	Ascospores	Phialides	Conidia	Reference
*O.nujiangensis*	Hepialidae larvae	Solitary, 148–182 mm long	–	–	–	54.9–76.5 µm long, base width 3.6–4.9 µm, tip width 1.0–1.5 µm	Oval or fusiform, 6.4–11.2 × 3.7–6.4 µm	This study
*O.karstii*	On dead larva of *Hepialusjianchuanensis*	Mostly single, 140–145 × 2–4 mm	Superficial, flask-shaped, 600–765 × 247–323 μm	Narrow cylindrical, 186–228 × 8–12 μm	Fusiform, 173–202 × 3–5 μm, not breaking into secondly spores	–	–	Li et al. (2016)
*O.liangshanensis*	Hepialidae larvae	Single or occasionally, 200–300 × 1.5–2.5 mm	Superficial, long ovoid, 450–740 × 300–450 μm	Cylindrical, 260–480 × 8–12 μm	Fasciculate, thread-like, slender, and long, 170–240 × 2.5–4.1 μm	Monophialidic, 46.9–75.6 μm long, subcylindrical, 3.8–4.7 μm basal wide	Ellipsoid, citriform or shape of an orange segment, 8.0–12.6 × 3.6–5.0 μm	Wang et al. (2021)
*O.sinensis*	Hepialidae larva	Single, occasionally 2–3, 40–110 mm long	Nearly superficial, ellipsoidal to ovate, 380–550 × 140–240 μm	Slender, long, 240–485 × 12–16 μm	Usually 2–4 mature ascospores, multiseptate, not breaking into secondary ascospores, 160–470 × 5–6 μm	–	–	Liang et al. (2007)

### ﻿Fungal community composition

In total, 135,048 effective sequences were obtained. Based on the minimum number of reads in the sample, 33,762 reads were randomly selected for each sample to avoid bias in the sequencing depth. The rarefaction curve (the Shannon-Wiener curve) showed that the sequencing depth was very reasonable for representing the diversity of the fungal community (Suppl. material 3).

**Figure 4.**
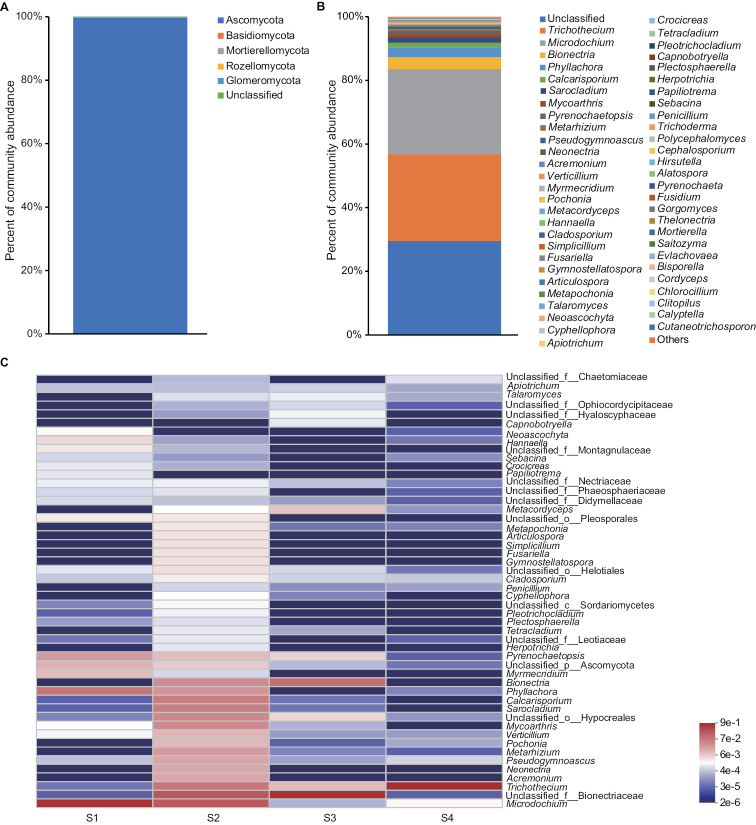
Composition of fungal community inhabiting *Ophiocordycepsnujiangensis*. A Composition of fungal community on phylum level. B Composition of fungal community on genus level. C Community heatmap analysis of the four specimens on genus level.

At the phylum level, a total of five phyla were identified, including Ascomycota, Basidiomycota, Mortierellomycota, Rozellomycota, and Glomeromycota. Of these, Ascomycota was dominant, with an average of 99.66%. The rest averaged no more than 1 percent. And the unclassified was dominant in the 151 identified genera, the average proportion was 29.56%, followed by *Trichothecium* (27.16%) and *Microdochium* (26.81%) (Fig. 4). Namely, numerous companion fungi were verified in the fruiting body of *O.nujiangensis*. The results also indirectly suggested that *O.nujiangensis* might be a new species as its ITS sequence could not be aligned in the database.

### ﻿Phylogenetic analyses of the fungi at the family level

The top 50 families were classified into four phyla (Suppl. material 4), comprising Ascomycota, Basidiomycota, Mortierellomycota, and Rozellomycota; however, none in Glomeromycota. There were 41 families subordinated to Ascomycota, including the three families (Clavicipitaceae, Ophiocordycipitaceae, and Cordycipitaceae), which distributed *Cordycepssensu lato.* And the phylogenetic locations of the three families were essentially the same as previously reported in the study by Sung et al. (2007) and Wang et al. (2020a). The results implied that *O.nujiangensis* might have many companion fungi, which belongs to *Cordycepssensu lato.*

## ﻿Discussion

*Ophiocordycepsnujiangensis* was morphologically characterized by solitary, woody, and dark brown stromata, smooth-walled and septate hyphae, solitary and gradually tapering conidiogenous cells with plenty of warty protrusions, and oval or fusiform conidia with mucinous sheath. In this research, the five-gene phylogenetic tree was rebuilt with four clades of *Ophiocordyceps* (the clade of *Hirsutella*, the clade of *O.ravenelii*, the clade of *O.sobolifera*, and the clade of *O.sphecocephala*,) and six subclades of *Hirsutella* clade (the subclade of *H.citriformis*, the subclade of *H.guyana*, the subclade of *H.nodulosa*, the subclade of *H.sinensis*, the subclade of *H.thompsonii*, and the subclade of *Hirsutella* ant pathogen), and the results were similar to the analyses by Sanjuan et al. (2015), Simmons et al. (2015), and Wang et al. (2018). *O.nujiangensis* was grouped phylogenetically with *O.karstii*, *O.liangshanensis*, and *O.sinensis*. Nevertheless, there was an obvious distinction between them in their morphological characteristics, especially in the length of the stromata. However, further comparisons were difficult due to the lack of anamorph observation of *O.karstii*. In the phylogenetic analyses of nuclear genes, the three specimens of *O.nujiangensis* united to form a single clade, and the result of phylogenetic analysis was consistent with that based on mitochondrial genes. Not only that, metagenomic data of *O.karstii* and *O.liangshanensis* had not been reported, and the differences between the allied species could not be discriminated.

A total of five fungal phyla and 151 fungal genera were identified in this study. Among them, Ascomycota and the unclassified were the dominant phylum and genus. Except for the dominant, *Trichothecium* and *Microdochium* also had high proportions at the genus level. The genus, *Trichothecium*, was a heterogonous group of filamentous fungi; some species were pathogenic fungi (Summerbell et al. 2011; Han et al. 2021). *Microdochium* was a common cereal pathogen fungus that adapted nicely to the cool (Parry et al. 1995; Gagkaeva et al. 2020). Some companion fungi had been confirmed that had vital functions (Guo et al. 2002; Li et al. 2018). The growth and development of the host were mostly due to the combined effect of the microbial adding peptone and yeast community (Han et al. 2019; Xie et al. 2021). Thus, the genera might have had an essential influence on the growth and development of *O.nujiangensis*. Furthermore, a comparison of the fungal communities of *O.sinensis* and *O.nujiangensis* showed that they had different community compositions. However, *Trichothecium* and *Microdochium* could not be found among the top 19 genera in fungal communities of *O.sinensis* reported (Xia et al. 2016). Consequently, the differences in the microbial community composition of closely related species might be suitable as further evidence for identifying species.

The phylogenetic analysis of mitochondrial genes became an adequate means to delimit fungal species, except for morphological observation and the five-gene phylogenetic tree (Nie et al. 2019; Meng et al. 2020). Similar topologies were obtained by utilizing 14 PCGs, PCGs + rRNA, or mitochondrial whole genomes (Hu et al. 2021). It was illustrated that the stable phylogenetic trees could be reconstructed using the phylogenetic analysis of mitochondrial genes. In the present research, the phylogenetic tree of Hypocreales was rebuilt, which was similar to the report by Chen et al. (2021). It had been shown that the phylogenetic trees with mitochondrial genes were reliable.

The characteristic differences between the new species and other species could be distinguished through the morphology data, and the phylogenetic location of the new species could be determined by the phylogeny and mitogenomics data. It was attempted to further study the companion fungi of the new species, but the available data on the species and their phylogenetic relationship were considerably lacking. Metagenomics provided more comprehensive genetic information about microorganisms and the microorganisms with which they associated (Venter et al. 2004; Truong et al. 2017; Huang and Wang 2020). Therefore, the method might be an efficient avenue for reconstructing the “Tree of Life”.

## Supplementary Material

XML Treatment for Ophiocordyceps nujiangensis

## Supplementary materials

Supplementary material 1 Relevant species information and GeneBank accession numbers for phylogenetic research in this study Data type table (word document) https://binary.pensoft.net/file/785610This dataset is made available under the Open Database License (http://opendatacommons.org/licenses/odbl/1.0/). The Open Database License (ODbL) is a license agreement intended to allow users to freely share, modify, and use this Dataset while maintaining this same freedom for others, provided that the original source and author(s) are credited.Tao Sun, Weiqiu Zou, Quanying Dong, Ou Huang, Dexiang Tang, Hong Yu

Supplementary material 2 The information of species and their mitochondrial genomes for constructing the mitochondrial-genome phylogenetic tree of Hypocreales Data type table (PDF file) https://binary.pensoft.net/file/785611This dataset is made available under the Open Database License (http://opendatacommons.org/licenses/odbl/1.0/). The Open Database License (ODbL) is a license agreement intended to allow users to freely share, modify, and use this Dataset while maintaining this same freedom for others, provided that the original source and author(s) are credited.Tao Sun, Weiqiu Zou, Quanying Dong, Ou Huang, Dexiang Tang, Hong Yu

Supplementary material 3 Rarefaction curves (Shannon-Wiener curve) of the fungal communities collected from the fruiting body from four different specimens of *Ophiocordycepsnujiangensis* Data type figure (eps. file) https://binary.pensoft.net/file/785612This dataset is made available under the Open Database License (http://opendatacommons.org/licenses/odbl/1.0/). The Open Database License (ODbL) is a license agreement intended to allow users to freely share, modify, and use this Dataset while maintaining this same freedom for others, provided that the original source and author(s) are credited.Tao Sun, Weiqiu Zou, Quanying Dong, Ou Huang, Dexiang Tang, Hong Yu

Supplementary material 4 Phylogenetic analyses of the ranked top 50 families identified from *Ophiocordycepsnujiangensis* based on maximum likelihood (ML). Values at the nodes are ML bootstrap proportions Data type figure (PDF file) https://binary.pensoft.net/file/785613This dataset is made available under the Open Database License (http://opendatacommons.org/licenses/odbl/1.0/). The Open Database License (ODbL) is a license agreement intended to allow users to freely share, modify, and use this Dataset while maintaining this same freedom for others, provided that the original source and author(s) are credited.Tao Sun, Weiqiu Zou, Quanying Dong, Ou Huang, Dexiang Tang, Hong Yu
