# Supplementary material for: ﻿Morphology, phylogeny, mitogenomics and metagenomics reveal a new entomopathogenic fungus Ophiocordycepsnujiangensis (Hypocreales, Ophiocordycipitaceae) from Southwestern China
Source: MycoKeys. 2022 Dec 21;94:91–108. doi: 10.3897/mycokeys.94.89425 (PMC9836510; doi:10.3897/mycokeys.94.89425)
Supplement: Supplementary material 2 — The information of species and their mitochondrial genomes for constructing the mitochondrial-genome phylogenetic tree of Hypocreales [file mycokeys-94-091-s002.pdf]

Table 2 The information of species and their mitochondrial genomes for constructing the mitochondrial-genome phylogenetic tree of Hypocreales

Order	Family	Species	Genbank ID	Strain	Notes
Eurotiales	Aspergillaceae	*Penicillium citrinum*	NC047444	JH1205	Outgroup
Sordariales	Sordariaceae	*Neurospora crassa*	KY498478	FGSC 9718	
Hypocreales	Bionectriaceae	*Clonostachys rosea*	NC036667	6792	
		Acremonium chrysogenum	NC023268	ATCC:11550	
		Acremonium fuci	NC029851	3a34	
		Clonostachys compactiuscula	MW030498	YFCC 897	
		Clonostachys rogersoniana	MW030499	YFCC 899	
	Hypocreaceae	*Paecilomyces penicillatus*	NC043850	SAAS_ppe1	
		Trichoderma hamatum	NC036144	—	
		Hypomyces aurantius	NC030206	—	
		Trichoderma asperellum	NC037075	B05	
		Trichoderma reesei	NC003388	QM9414	
	Ophiocordycipitaceae	***Ophiocordyceps nujiangensis***		**YFCC8894**	Material examined
		Tolypocladium cylindrosporum	NC046839	ARSEF963	
		Tolypocladium inflatum	KY924883	ARSEF 616	
		Tolypocladium ophioglossoides	NC031384	L2	
		Tolypocladium guangdongense	NC054274	GD15	
		Ophiocordyceps sinensis	NC034659	CCTCC AF 2017003	
		Hirsutella rhossiliensis	NC030164	USA-87-5	
		Hirsutella vermicola	NC036610		
		Ophiocordyceps pingbianensis	MW042690		

	Hirsutella minnesotensis	NC027660	
	Hirsutella thompsonii	NC040165	
Clavicipitaceae	*Epichloe hybrida*	KX066187	Lp1
	Epichloe festucae	NC032064	AR5
	Epichloe typhina	NC032063	E8
	Metarhizium album	MW448543	ARSEF1941
	Metarhizium brunneum	LR792747	ARSEF 4556
	Metarhizium rileyi	MT107156	RCEF 4871
	Metarhizium robertsii	JELW01000367	ARSEF 2575
	Orbiocrella petchii	MT447058	SD3
	Metacordyceps chlamydosporia	NC022835	170
Cordycipitaceae	*Samsoniella hepiali*	KJ764671	—
	Lecanicillium saksenae	NC028330	CGMCC5329
	Akanthomyces lecanii	NC046840	RCEF1005
	Beauveria bassiana	NC010652	Bb13
	Beauveria malawiensis	NC030635	k89
	Beauveria pseudobassiana	NC022708	C1010
	Beauveria caledonica	NC030636	fhr1
	Beauveria brongniartii	NC011194	IMBST95031
	Cordyceps militaris	NC022834	EFCC-C2
	Cordyceps pruinosa	MN515031	CP1
	Cordyceps cicadae	NC041489	CCAD02
	Cordyceps chanhua	MH734937	JGS-7
	Cordyceps	MK234910	YFCC

	tenuipes		2017002
	Cordyceps farinosa	OM201302	YFCC8744
	Parengyodontium album	KX061492	ATCC:56482
Nectreaceae	*Fusarium proliferatum*	LT841261	ITEM2400
	Fusarium oxysporum	NC017930	F11
	Fusarium gerlachii	NC025928	CBS 123666
	Fusarium fujikuroi	JX910420	IMI58289
	Fusarium culmorum	NC026993	CBS 139512
	Fusarium commune	NC036106	JCM11502
	Fusarium solani	NC016680	mpVI
	Fusarium verticillioides	NC016687	7600
	Ilyonectria destructans	NC030340	2007/P/476
