# Supplementary figures and images for: ﻿Morphology, phylogeny, mitogenomics and metagenomics reveal a new entomopathogenic fungus Ophiocordycepsnujiangensis (Hypocreales, Ophiocordycipitaceae) from Southwestern China
Source: MycoKeys. 2022 Dec 21;94:91–108. doi: 10.3897/mycokeys.94.89425 (PMC9836510; doi:10.3897/mycokeys.94.89425)

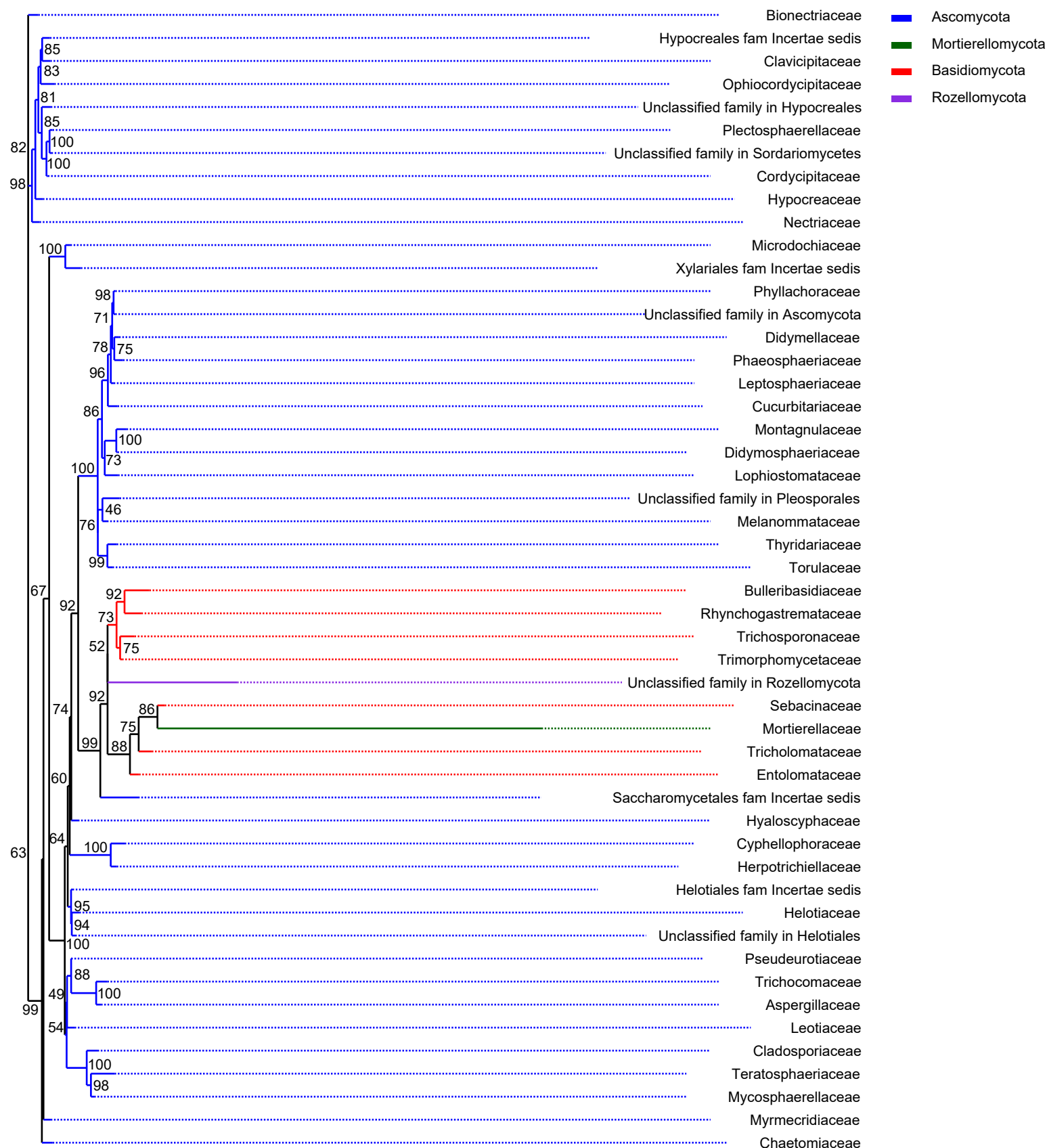

Supplement: Supplementary material 4 — Phylogenetic analyses of the ranked top 50 families identified from Ophiocordycepsnujiangensis based on maximum likelihood (ML). Values at the nodes are ML bootstrap proportions [file mycokeys-94-091-s004.pdf]
